# A Large Scale shRNA Barcode Screen Identifies the Circadian Clock Component ARNTL as Putative Regulator of the p53 Tumor Suppressor Pathway

**DOI:** 10.1371/journal.pone.0004798

**Published:** 2009-03-11

**Authors:** Jasper Mullenders, Armida W. M. Fabius, Mandy Madiredjo, René Bernards, Roderick L. Beijersbergen

**Affiliations:** Division of Molecular Carcinogenesis, Centre for Biomedical Genetics and Cancer Genomics Centre, Netherlands Cancer Institute, Amsterdam, The Netherlands; The University of Hong Kong, Hong Kong

## Abstract

**Background:**

The p53 tumor suppressor gene is mutated in about half of human cancers, but the p53 pathway is thought to be functionally inactivated in the vast majority of cancer. Understanding how tumor cells can become insensitive to p53 activation is therefore of major importance. Using an RNAi-based genetic screen, we have identified three novel genes that regulate p53 function.

**Results:**

We have screened the NKI shRNA library targeting 8,000 human genes to identify modulators of p53 function. Using the shRNA barcode technique we were able to quickly identify active shRNA vectors from a complex mixture. Validation of the screening results indicates that the shRNA barcode technique can reliable identify active shRNA vectors from a complex pool. Using this approach we have identified three genes, *ARNTL*, *RBCK1* and *TNIP1*, previously unknown to regulate p53 function. Importantly, *ARNTL* (*BMAL1*) is an established component of the circadian regulatory network. The latter finding adds to recent observations that link circadian rhythm to the cell cycle and cancer. We show that cells having suppressed *ARNTL* are unable to arrest upon p53 activation associated with an inability to activate the p53 target gene *p21^CIP1^*.

**Conclusions:**

We identified three new regulators of the p53 pathway through a functional genetic screen. The identification of the circadian core component ARNTL strengthens the link between circadian rhythm and cancer.

## Introduction

The *TP53* gene product is important in the cellular response to different types of stress [1], [2]. A major physiological stress is DNA damage. DNA damage leads to activation of the ATM/ATR, CHK1/2 cascade, which in turn activates p53. Activation of p53 is achieved by increased stability and post translational modifications of the p53 protein. These modifications include phosphorylation, methylation [3], ubiquitination [4], [5] and acetylation [6] leading to enhanced transcriptional activity of p53. Furthermore, oncogene activation can also lead to p53 activation through activation of the p19^ARF^ protein. p19^ARF^ inhibits MDM2, the major E3 ubiquitin ligase for p53 [7] leading to stabilization and activation of p53. Activation of p53 leads to transcriptional activation of a large set of p53 target genes, which in turn causes cell cycle arrest or apoptosis [8].

The p53 pathway is inactivated in almost all human cancers [9]. In about half of human cancers this is due to mutation or deletion of the *TP53* gene itself. However in a significant fraction of human tumors, the p53 pathway is inactivated through alteration in cellular components acting up- or down-stream of p53. For example, amplification of the negative regulator of p53, *MDM2*, leads to accelerated degradation and inactivation of p53 [2], [10].

As a model to screen for genes that modulate p53 function, we previously developed a human fibroblast cell line named BJtsLT [11]. These cells express a temperature-sensitive mutant of the SV40 large T antigen, which allows the cells to proliferate at the permissive temperature (32°C). However, when the cells are shifted to 39°C, the large T antigen is degraded and the cells enter a stable p53-dependent cell cycle arrest.

We and others have previously described the construction and initial screening of shRNA libraries using the barcode technique [11]–[17]. The barcode technique allows the rapid identification of individual shRNA vectors from a large pool of shRNA vectors that produce a specific phenotype. This approach takes advantage of the fact that each shRNA vector contains a unique 19-mer sequence as part of the shRNA cassette, which can serve as a molecular “barcode” identifier. Briefly, cells are infected with the pooled shRNA library of some 24,000 vectors. The population of cells is then split into two separate populations. One population is used as a reference sample while the other sample is subjected to a selective treatment. Knockdown of a specific gene by the shRNA vector can lead to three possible cellular responses to this treatment. First the cells can remain unaffected identical to control cells, second the cells can become more sensitive to the treatment and third the cells can acquire resistance to the treatment. As a consequence of the differential response to the treatment, the relative number of cells that harbor a specific shRNA can increase, decrease or remain the same. The relative abundance of each shRNA cassette can be determined by the isolation of the shRNA cassettes from the population, labeling of the barcode identifiers with different fluorescent dyes and subsequent hybridization to DNA microarrays representing all shRNA sequences. By comparing the relative abundance of all shRNAs against the reference population, shRNAs responsible for the three possible phenotypes can be identified.

The shRNA barcode technique can be used for different screening approaches, most notably the identification of genes that are involved in drug resistance. For instance, we have used the barcoding approach to identify genes that are involved in the resistance to the cytotoxic effects of Nutlin-3, a drug that activates p53 by acting as an inhibitor of MDM2 [13]. This led to the identification of 53BP1, a p53 binding protein, as a critical modulator of the effect of Nutlin-3. More recently, we found the tumor suppressor *PTEN* as a gene, which upon decreased expression confers resistance to trastuzumab in breast cancer [12]. Significantly, the PI3K pathway, which is negatively regulated by PTEN, was also shown to be a major regulator of trastuzumab sensitivity in the clinic, underscoring the utility of the *in vitro* genetic screens to identify drug modulators.

In the first screen applying the NKI shRNA library, Berns et al. used BJtsLT cells to identify 5 new players in the p53 pathway [11]. This screen did not take advantage of the barcode technology. Rather, it was performed by the conventional method of isolating and expanding colonies that are resistant to p53-mediated growth arrest. Subsequent isolation of the shRNA inserts and sequence analysis was required to identify the shRNA responsible for the bypass of the p53 mediated cell cycle arrest. This method is rather laborious and such an approach may not uncover all active shRNAs in a library. Moreover, due to the labour-intensive nature of the screen, only part of the 24,000 vector NKI shRNA library was covered in this initial approach. Here we describe the screening of the entire NKI shRNA library for modulators of p53 activity using the barcode technology. We find three additional genes whose suppression causes resistance to p53-dependent proliferation arrest.

## Results

### Screening of the BJtsLT cells

**Figure 1:**
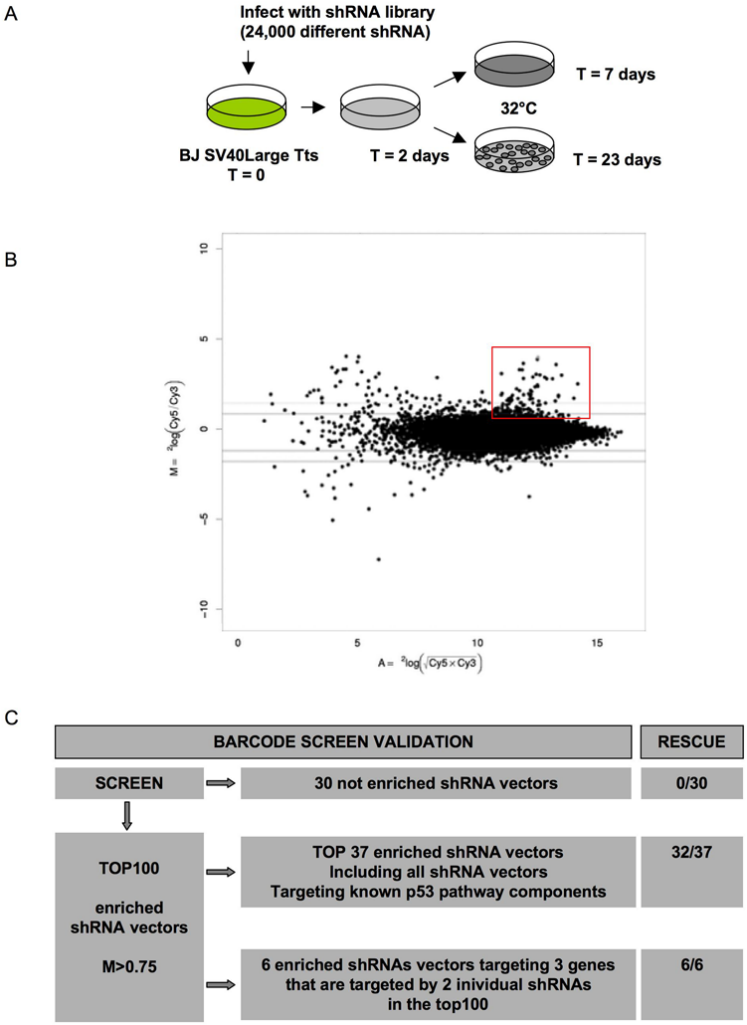
shRNA barcode screen identifies mediators of the p53 dependent cell cycle arrest. a) Schematic outline of the BJtsLT genetic screen. BJtsLT cells were infected with the NKI shRNA library and were either left at 32°C or shifted to 39°C. After 7 days the cells at 32°C had reached confluency and were harvested. Cells at 39°C were harvested after 23 days after which they had formed visible colonies. b) Analysis of the relative abundance of shRNAs recovered from the BJtsLT barcode experiment. Data are normalized and plotted as *M*, the ^2^log (ratio Cy5/Cy3), versus *A* (^2^log(√intensity Cy3×Cy5)). The data are the average of two independent hybridization experiments performed in duplicate with reversed colour. A red box is drawn around the top 100 enriched shRNAs at 39°C. c) Schematic overview of selection criteria used to select hits from the shRNA barcode screen for further validation.

To identify shRNA vectors that can modulate the activity of the p53 pathway, we performed a shRNA bar code screen in BJtsLT cells infected with the NKI shRNA library (See Figure 1a). These cells proliferate at 32°C but enter into a p53-dependent proliferation arrest at 39°C [11]. The infected cells were cultured for three days at 32°C to allow retroviral integration and for gene knockdown to become effective. At day 4 the cells were split into two populations; one was kept at 32°C and the other was shifted to 39°C. The BJtsLT at 32°C were cultured until the population reached confluency and genomic DNA was isolated. The BJtsLT cells grown at 39°C cease proliferation unless a shRNA is expressed that inactivates the p53-dependent anti-proliferative response. Such cells will continue to proliferate and give rise to a colony. In addition to infection with the NKI shRNA library, we also used a shRNA vector targeting p53 as a positive control for colony outgrowth of the BJtsLT cells at 39°C. After 3 weeks of culturing at 39°C, the control plates infected with p53 shRNA vector contained large numbers of colonies consisting of rapidly proliferating cells (data not shown). The plates infected with the shRNA library also contained several colonies. Colonies from the library-infected plates were pooled and genomic DNA was isolated. The shRNA cassettes were recovered from the genomic DNA using PCR. The recovered shRNA inserts from both the control population and the colonies that continued proliferation at 39°C following infection with the shRNA library, were used to hybridize DNA microarrays containing all 24,000 19-mer sequences of the NKI shRNA library. The hybridization was performed for each of the replicate experiments, after which the results were combined to increase the statistical significance of the enriched shRNA vectors identified. Those shRNA vectors that confer resistance to the p53 mediated cell cycle arrest are enriched in the population cultured at 39°C and can be detected as outliers in a MA-plot representation of the barcode microarray experiment (figure 1b).

From this MA-plot we generated a “hit list” of shRNA vectors that are specifically enriched in cell cultured at 39°C. The following considerations were used to produce this hit list (summarized in figure 1c). We excluded all shRNAs that had an intensity (A-value) lower than 10, as spots with a relative low intensity are likely to be “noise” and as a consequence can have aberrantly high “M” ratios. In addition we only included shRNAs if their ratio (M = 2log ratio cy5/cy3) was >0.75, this effectively means the top 100 most enriched shRNAs on the micro-array.

To shorten the list of shRNA vectors to be validated, we first asked where shRNA vectors targeting known components of the p53 pathway were positioned in the top 100. In total, five shRNA vectors targeting known components of the p53 pathway were present in the top 100 and their distribution was as follows (Table 1): one shRNA vector targeting p53 (position #8), two shRNA vectors targeting p21^cip1^ (#7 & #37) and two shRNA vectors targeting 53BP1 (#5 & #36). As we identified shRNA vectors targeting 53BP1 and p21^cip1^ at positions 36 and 37 on the hit list, we decided to individually test all shRNA vectors on positions 1–37 in a second round selection. Included in this set are vectors targeting 3 out of 5 genes that were previously identified and validated by Berns et al., (2004): *HDAC4* (#2) *KIA0828* (#13), *HTATIP* (#25). The identification of these shRNA vectors provides further support for the notion that the barcode method enables both fast and reliable screening of shRNA libraries.

**Table 1:** List of shRNA vectors that were selected by the criteria as in figure 1c. Depicted are shRNAs that were used in validation experiments.

Rank	M	A	Rescue	HUGO	RefSeq	19mer_start	19mer_sequence	Remarks
1	4.07	12.24	+	KCNK9	NM_016601	217	GTACAACATCAGCAGCGAG	
2	3.95	12.22	+	HDAC4	NM_006037	3751	GCATGTGTTTCTGCCTTGC	Berns et al
3	3.73	11.65	+	ABHD6	NM_020676	374	GGATATGTGGCTCAGTGTG	
4	3.67	12.98	+	MYCL1	NM_005376	933	GAGACACTCCAAACCTGAA	
5	3.39	11.49	+	TP53BP1	NM_005657	657	GATACTGCCTCATCACAGT	Known p53 pathway
6	3.39	11.60	+	XRCC1	NM_006297	258	GGAGGAGCAGATACACAGT	
7	3.18	10.76	+	CDKN1A	NM_078467	919	CTAGGCGGTTGAATGAGAG	Known p53 pathway
8	3.14	12.17	+	TP53	NM_000546	1026	GACTCCAGTGGTAATCTAC	Known p53 pathway
9	3.09	13.20	+	CALCA	NM_001741	428	AGGGATATGTCCAGCGACT	
10	3.00	12.03	+	RBCK1	NM_031229	1389	GTCAGTACCAGCAGCGGAA	this manuscript
11	2.99	12.52	+	INSRR	J05046	2402	GAACAGTGTCCTTCTGCGC	
12	2.92	11.65	+	SSR4	NM_006280	377	GAGTCCTACAGCCTCCTCA	
13	2.91	12.60	+	KIAA0828	NM_015328	3945	GAGTACATTCTGCCTTGCT	Berns et al
14	2.91	12.83	+	PTPRN2	NM_002847	2968	GAGATTGATATCGCAGCGA	
15	2.63	13.86	+	TCEAL1	NM_004780	386	GGACCTGTTTGAGGTTCGC	
16	2.56	12.22	+	LHX3	AF156888	266	GTGTCTCAAGTGCAGCGAC	
17	2.47	12.12	+	NR2E3	AF148128	852	GTGGGCCAAGAACCTGCCT	
18	2.35	11.37	+	LOC90925	BC002792	226	AGAACTGGAGTGGATGGGG	
19	2.28	11.94	+	MPZ	NM_000530	793	GGATAAGAAATAGCGGTTA	
20	2.26	11.49	+	NR2E3	NM_014249	889	GTGGGCCAAGAACCTGCCT	
21	2.21	11.98	+	PENK	NM_006211	779	GATACGGAGGATTTATGAG	
22	2.12	10.78	+	SLIT2	NM_004787	490	AGAGGAGCATTCCAGGATC	
23	2.09	11.87	+	TRAR3	NM_175057	52	GTGAACGAATCCTGCATTA	
24	2.04	11.66	−	CDKN2A	NM_000077	745	GAACCAGAGAGGCTCTGAG	
25	2.04	11.96	+	HTATIP	NM_006388	1045	GTACGGCCGTAGTCTCAAG	Berns et al
26	2.02	11.21	+	PRPF18	NM_003675	228	AGAGGAGGACCAGAAACCA	
27	2.02	11.93	−	GOLGA5	NM_005113	2209	GATACCCCATAGCGCGAGT	
28	2.01	13.08	−	TIEG	NM_005655	2179	GAATTGGAATCCTCCTTAA	
29	1.97	11.92	−	COL12A1	NM_004370	1844	GGATGCCGTTCGCTCAGAA	
30	1.94	13.07	+	RAD51C	NM_058216	263	GATATGCTGGTACATCTGA	
31	1.85	13.70	−	TLR4	NM_003266	2180	GACCATCATTGGTGTGTCG	
32	1.83	11.43	+	RAB2	NM_002865	725	GAAGGAGTCTTTGACATTA	
33	1.83	11.05	+	ZNF347	NM_032584	387	GAGTAATACAGGAGAAGTA	
34	1.82	12.01	+	HSD17B4	NM_000414	142	AGAGGAGCGTTAGTTGTTG	
35	1.80	11.91	−	GCGR	NM_000160	547	AGTGCAACACCGCTTCGTG	
36	1.75	11.19	+	TP53BP1	NM_005657	387	GAACGAGGAGACGGTAATA	Known p53 pathway
37	1.72	11.09	+	CDKN1A	NM_078467	560	GACCATGTGGACCTGTCAC	Known p53 pathway
40	1.67	11.13	+	RPS6KA6	NM_014496		GATTATCCAAAGAGGTTCT	Berns et al
46	1.49	10.96	+	RBCK1	NM_031229	710	GGGGATGAACAGGTGGCAA	this manuscript
51	1.31	10.57	+	TNIP1	NM_006058	1718	GGAAGAGCTGAAGAAGCAA	this manuscript
59	1.23	11.67	+	TNIP1	NM_006058	408	GAGTCCCAGATGGAAGCGA	this mansucript
74	1.00	10.42	+	ARNTL	NM_001178	1468	GAACTTCTAGGCACATCGT	this manuscript
99	0.76	11.55	+	ARNTL	NM_001178	590	GGGAAGCTCACAGTCAGAT	this manuscript

Under rescue; + means a validated shRNA, − means not validated. *M* indicates the ^2^log (ratio Cy5/Cy3), A the ^2^log(√intensity Cy3×Cy5).

In addition, we selected 3 genes that were represented by two independent shRNAs in the top 100 (of which one is present in the 37 already selected shRNAs), bringing the total number of shRNAs to be tested to 42. We included these 3 additional genes in the validation, because if two independent shRNAs targeting the same transcript are enriched in the shRNA screen, this gives higher level of confidence to that specific hit. This is because it is less likely to be “off target” when two independent shRNAs yield the same phenotype and such off target effects of shRNAs are a common problem in these types of genetic screens [18], [19]. The other three genes, *ARNTL* [*20*], *RBCK1* [*21*] and *TNIP1* [*22*] have not been linked to p53 before.

**Figure 2:**
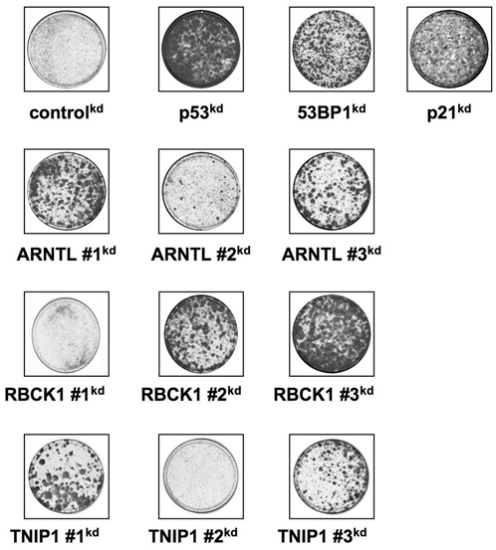
Colony formation ARNTL, RBCK1 and TNIP1 shRNA vectors. Cells were infected with shRNA vectors targeting ARNTL, RBCK1 and TNIP1 and control shRNA vectors targeting GFP, p53, 53BP1 and p21. Cells were infected at 32°C and shifted to 39°C 2 days after infection. After three weeks culture at 39°C, the cells were fixed and stained.

To prove that the shRNA barcode technique specifically identifies shRNAs that are enriched in the experiment we also tested 30 randomly-selected shRNAs not enriched in the experiment. All shRNAs were re-tested in the BJtsLT cells. Cells were infected with individual shRNAs, shifted to 39°C and incubated for 3 weeks. When colonies were observed the cells were fixed and stained. As expected, none of the 30 randomly-selected shRNAs was able to produce colonies at 39°C (data not shown). However, for 37 of the 42 enriched shRNA vectors, we could clearly demonstrate that they allowed the cells to proliferate at 39°C. Among the genes targeted by these 37 shRNA vectors were all known p53 pathway components. In addition, we also observed rescue of growth arrest by all three genes that were targeted by two individual shRNAs: *ARNTL*, *RBCK1 and TNIP1 (* *Figure 2* *)*. Therefore we decided to focus on these three newly identified genes for which we identified two independent shRNAs.

To show the sensitivity and selectivity of the shRNA barcode technique, we decided to test all three shRNA vectors targeting *ARNTL*, *RBCK1* and *TNIP1* that are present in the library. As mentioned before, for these three genes we found only two of the three shRNAs to be enriched in the screen, whereas one shRNA vector was not enriched. When we infected all three shRNA vectors independently into BJtsLT cells we found that only shRNAs enriched in the shRNA barcode screen gave rise to colonies (Figure 2).

### Knockdown of target genes by shRNA vectors

**Figure 3:**
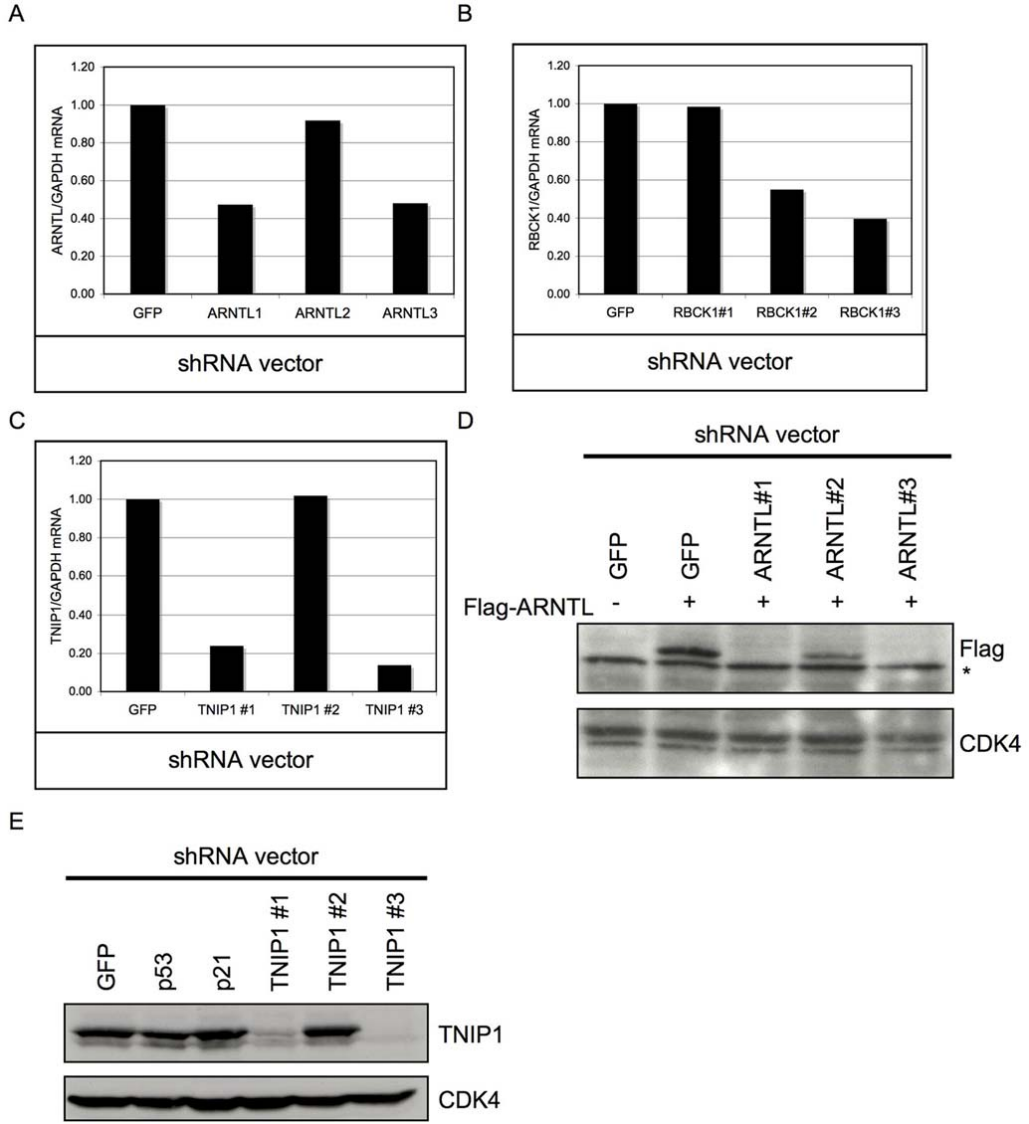
Barcode identified shRNA vectors suppress protein and mRNA levels of their targets. a) QRT-PCR for ARNTL in BJtsLT cells. BJtsLT cells were infected with indicated shRNA vectors. Samples for RNA isolation were taken 8 days after shift to 39°C. b) QRT-PCR for RBCK1 in BJtsLT cells. BJtsLT cells were infected with indicated shRNA vectors. Samples for RNA isolation were taken 8 days after shift to 39°C. c) QRT-PCR for TNIP1 in BJtsLT cells. BJtsLT cells were infected with indicated shRNA vectors. Samples for RNA isolation were taken 8 days after shift to 39°C. d) Flag-ARNTL together with the shRNA vectors targeting ARNTL were transiently transfected in Phoenix cells. Extracts were immunoblotted using Flag and CDK4 (control) antibodies. e) BJ cells were infected with the indicated shRNA vectors and Extracts were immunoblotted using TNIP1 and CDK4 (control) antibodies.

Next, we investigated if the shRNA vectors targeting *ARNTL*, *TNIP1* and *RBCK1* also reduced mRNA levels of their cognate target genes. BJtsLT cells were infected at 32°C and shifted to 39°C 3 days after infection. When colonies appeared, RNA was isolated and subjected to quantification by QRT-PCR. The result from the QRT-PCR showed that enriched shRNA vectors targeting ARNTL, RBCK1 and TNIP1 were more potent in decreasing target mRNA than the shRNA vectors that were not enriched (Figure 3a, 3b, 3c). In addition, we tested for both ARNTL and TNIP1 if protein levels were also affected by the shRNA vectors. For ARNTL we co-expressed the three shRNA vectors together with a cDNA encoding hARNTL in Phoenix cells. From the western blot analysis it was clear that only the vectors that could produce colonies also induced potent knockdown of protein expression, thus linking gene knockdown to the p53 growth arrest bypass phenotype (Figure 3d). Knockdown of TNIP1 was determined by analyzing endogenous protein levels in BJtsLT cells (Figure 3e). As can be seen only the vectors that are enriched in the barcode screen and validated to enable colony growth at 39°C were able to reduce endogenous TNIP1 protein levels. We conclude that by limiting the hit selection to genes that are targeted by two independent shRNAs we have only selected ‘on-target’ hits from a complex library.

### Knockdown of ARNTL, RBCK1 and TNIP1 in BJtsLT cells leads to reduced p21^CIP1^ levels

**Figure 4:**
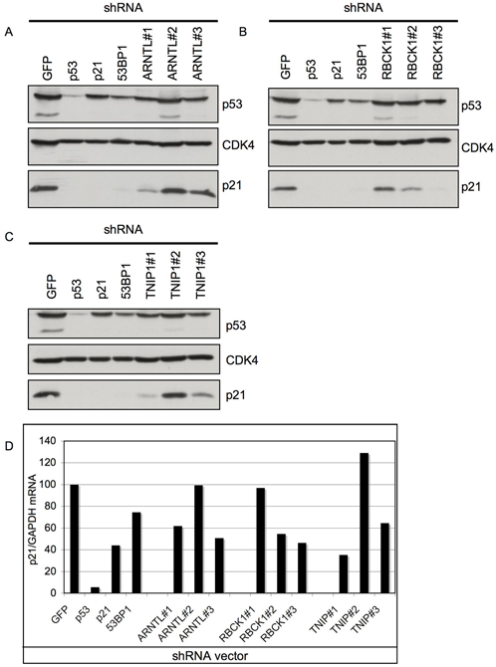
Knockdown of ARNTL, TNIP1 and RBCK1 prevents p21^CIP1^induction in BJtsLT cells. BJtsLT cells were infected at 32°C and shifted to 39°C for colony formation. After 14 days of culturing at 39°C cells were harvested, protein lysates were prepared and subjected to western blot for p53, CDK4 (control) and p21^CIP1^ (Figure 4a–c). Additionally, total RNA was isolated and used for QRT-PCR for p21^CIP1^ (Figure 4d).

*p21^CIP1^* is one of the critical effectors of p53 to induce a cell cycle arrest [23]. This is further supported by our identification of two shRNAs targeting *p21^CIP1^* in the list of outliers of the BJtsLT screen. We therefore examined the effect of knockdown of *ARNTL*, *RBCK1* and *TNIP1* on *p21^CIP1^* induction. We tested the effects on p21^CIP1^ in the BJtsLT system that we used for the initial screen. When BJtsLT cells are shifted to 39°C, a rapid increase in p21^CIP1^ protein levels is observed (Figure 4a). As expected, this p21^CIP1^ induction is attenuated in cells infected with shRNA vectors targeting *p53*, *p21^CIP1^* or *53BP1* (Figure 4a). When we used shRNA vectors targeting *ARNTL*, *RBCK1* and *TNIP1* we observed a decrease in p21^CIP1^ levels for those shRNA vectors that produced colonies at 39°C, but not for shRNA vectors that failed to produce colonies (Figure 4a–c).

To be sure that the decrease in p21^CIP1^ protein levels were caused by decreased *p21^CIP1^*transcription we also measured *p21^CIP1^* mRNA levels by QRT-PCR (Figure 4d). All shRNAs that could produce colonies at 39°C also showed a decrease in *p21^CIP1^* mRNA. This result suggests that the knockdown of *ARNTL*, *RBCK1* and *TNIP1* leads to a decreased transcriptional activity of p53 towards its target *p21^CIP1^*.

Recently multiple reports have discussed the relationship between cancer and circadian rhythm [24]–[26]. ARNTL is a core component of circadian rhythm transcriptional machinery [20], [27]. ARNTL binds to CLOCK and together they regulate expression of 1,000s of genes in a circadian timing [28], [29]. Genes regulated in a circadian fashion are involved in cell cycle, detoxification and other processes [30]. Therefore we decided to test if ARNTL is involved in the regulation of p21^CIP1^ expression in other cell systems.

### Reduced *p21^CIP1^*activation after DNA damage in HCT116 cells with ARNTL knockdown

**Figure 5:**
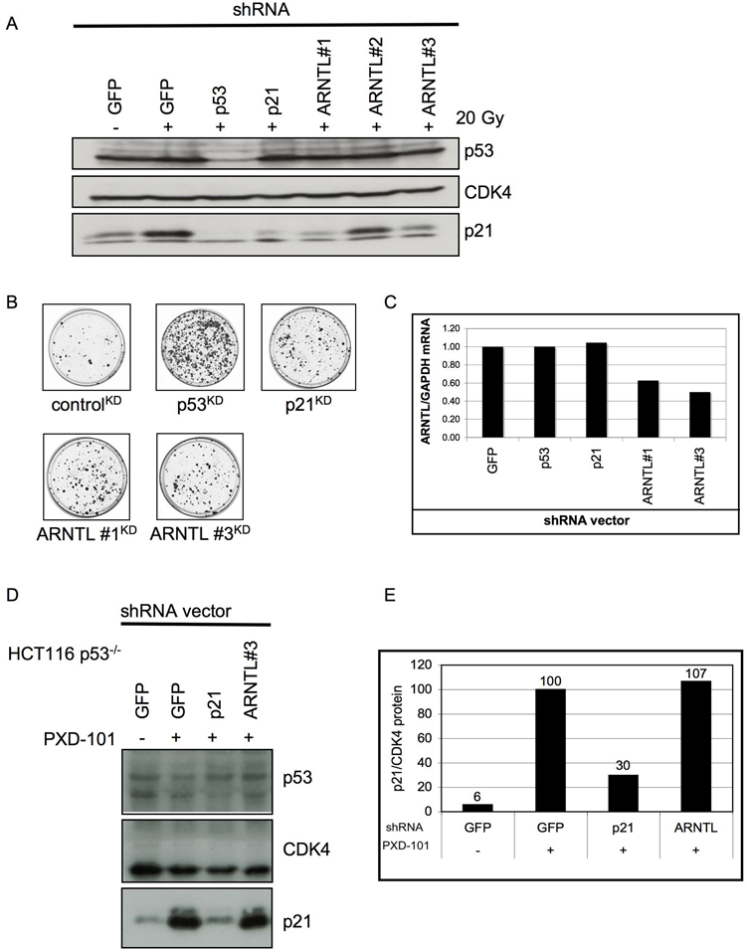
ARNTL regulates p21^CIP1^ expression. a) Knockdown of ARNTL inhibits radiation induced p21^CIP1^ induction. U2OS cells were infected with the shRNA vectors as indicated. Cells were seeded and irradiated with 20 Gy of γ-radiation. After o/n incubation cells were lysed and lysates were subjected to western blot using antibodies for p53, CDK4 (control) and p21^CIP1^. b) Knockdown of ARNTL can also rescue a p19-induced cell cycle arrest. U2OS cells were infected with the indicated shRNA vectors followed by a super-infection with p19ARF-RFP virus. Cells were seeded and incubated for three weeks. After three weeks the infected cells were fixed and stained. c) Knockdown of ARNTL in U2OS cells (Fig 5b) was quantified by QRT-PCR. d) ARNTL knockdown is not involved in p53 independent p21^CIP1^ induction. HCT116 wt and p53^−/−^ cells were infected with knockdown vectors targeting p53, p21^CIP1^ and ARNTL. Cells were treated with 0.5 µM PXD101 for 16 hrs. Cells were then lysed and lysates were subjected to western analysis for p53, CDK4 (control) and p21^CIP1^. e) Quantification of p21 protein levels in the western blot in figure 5c using IMAGE J software.

Normal human cells arrest either in G1 or S phase of the cell cycle after encountering DNA damage to repair the DNA, thereby preventing accumulation of mutations in the genome of daughter cells. The G1 phase cell cycle arrest is p53 dependent and mainly executed by the CDK inhibitor p21^CIP1^ [31], [32] . To investigate if ARNTL is also required for the *p21^CIP1^* activation after DNA damage, we infected U2OS osteosarcoma derived cells with different shRNAs targeting ARNTL. The cells were incubated to allow knockdown to take affect, after which cells were irradiated to inflict DNA damage and monitored for p21^CIP1^ activation. When we compared cells that were infected with a shRNA vector targeting p53 to cells infected with a control shRNA vector, we observed lower p53 and p21^CIP1^levels after γ-radiation. In the cells infected with ARNTL knockdown vectors we also observed lower p21^CIP1^protein levels, but p53 protein levels were unaffected (Figure 5a). This observation suggests that ARNTL can modulate the activity of p53 towards its target *p21^CIP1^*. However, we cannot distinguish between a specific effect of ARNTL on p21^CIP1^and a more general effect of ARNTL on p53 transcriptional activity.

### *ARNTL* knockdown also allows bypass a p19^ARF^ induced growth arrest

As the BJtsLT cells are quite artificial due to the presence of the SV40 T viral oncogene, we also investigated if *ARNTL* knockdown could bypass a more physiological p53-induced cell cycle arrest. To address this, we used cells in which we can activate p53 by over-expression of p19^ARF^. p19^ARF^ inhibits MDM2 function thereby leading to an increase of p53 protein and activation of target genes [33]. Activation of p19^ARF^ leads to a stable p53-dependent cell cycle arrest [34]. To test if *ARNTL* knockdown can also rescue a p19^ARF^-induced cell cycle arrest, we infected U2OS cells with the shRNAs targeting *ARNTL*. After knockdown had taken effect the cells were super-infected with a p19^ARF^ encoding retrovirus. We observed that cells with knockdown of *p53* or *p21^CIP1^* continue proliferation after the forced expression of *p19^ARF^* (Figure 5b), knockdown of *ARNTL* also allows cells to proliferate after p53 activation by p19^ARF^ (for knockdown see figure 5c). This result suggests that *ARNTL* expression is required for the anti-proliferative response of 19^ARF^ activation. When *ARNTL* levels are low, the cells escape this arrest.

### p53 independent *p21^CIP1^*activation does not require ARNTL

The results described above suggest a role for ARNTL in the regulation of *p21^CIP1^* expression by p53. However they do not rule out that ARNTL controls p21^CIP1^activation in a general fashion, independent of p53. To test this possibility we made use of small molecule HDAC inhibitors (HDACi). These HDACi cause induction of *p21^CIP1^* in both a p53 dependent and p53 independent manner [35]–[38]. In order to study the effect of ARNTL on p53 independent activation of p21^CIP1^, we made use of a HCT116 p53 knockout cell-line (HCT116 *p53* ^−/−^) and the HDACi PXD101 (Belinostat®) [32]. When these HCT116 p53^−/−^ cells are treated with PXD-101, a strong induction of p21^CIP1^ is observed (Fig 5d and e). HCT116 p53^−/−^ cells infected with a shRNA vector against *p21^CIP1^* show reduced p21^CIP1^ protein levels after PXD-101 treatment. However, cells infected with a shRNA targeting *ARNTL* do not show any alteration in the induction of p21^CIP1^ protein levels following HDACi treatment. Thus the p53-independent induction of p21^CIP1^ by HDACi is not dependent on ARNTL. From this we conclude that ARNTL is not generally required for *p21^cip1^* induction, but does affect the capacity of p53 to activate *p21^CIP1^* expression.

## Discussion

The screening of large-scale RNAi libraries has been used increasingly over the last years to identify the specific functions of genes in cellular pathways, networks and mechanisms. Here we describe the screening of a complex RNAi library to identify genes that were previously unknown to regulate a p53-dependent cell cycle arrest.

We have used the RNAi barcode technique to screen a human shRNA library containing ∼24,000 vectors targeting ∼8,000 genes. Using this approach, we were able to rapidly identify shRNAs that allow bypass of a p53 dependent cell cycle arrest. In total we confirmed that 32 out of the 37 genes that were identified by the barcode screen could indeed prevent cells from entering into a p53 dependent cell cycle arrest. However, only 5 of these 32 genes were targeted by two independent shRNAs. Two out of these five genes (TP53BP1 and *p21^cip1^*) are well-known to be involved in p53 signaling. However the other three genes (*TNIP1*, *RBCK1* and *ARNTL*) were previously not known to be involved in the p53 pathway.

The three newly identified genes all affect the induction of the p53 target gene p21^CIP1^ but no change in p53 protein stability is observed after ARNTL, TNIP1 or RBCK1 knockdown. Importantly, p21^CIP1^ knockdown alone is sufficient to rescue cells from the p53 induced cell cycle arrest. This observation indicates that the rescue of the p53 induced cell cycle arrest by ARNTL, RBCK1 or TNIP1 knockdown is the result of a lack of p21^CIP1^ induction by p53.

The activity of p53 has been mainly attributed to its role as transcription factor with tumor suppressive capacities. Therefore, we assessed if any of the genes identified in our screen had been linked to transcription before. TNIP1 was originally identified as an inhibitor of NF-κB signaling [22], [39]. Although it was shown that TNIP1 over-expression inhibits the transcriptional activity of the NF-κB heterodimer it is believed that this is an indirect effect through an currently unknown mechanism. The ubiquitin E3 ligase RBCK1 has been reported to regulate and ubiquitinate several proteins [21], [40]–[42]. Although experiments have been performed that suggest a role for RBCK1 in transcription [43] a clear role for RBCK1 in regulating transcription has not been reported up till now. This picture is different for ARNTL which is known to be the central transcription factor in regulating circadian rhythm. The critical role of ARTNL in circadian rhythm was demonstrated by the construction of the knockout mouse. Mice that are deficient for *ARNTL* are unable to maintain a circadian rhythm in constant darkness [20]. In addition, the *ARNTL* knockout mouse also suffers from premature aging [44]. In recent years, many other processes have been shown to be regulated in a circadian fashion. Most importantly it was shown that the mammalian cell cycle is controlled by circadian rhythm [45]. The possible involvement of circadian rhythm in cancer results from studies of the Period 2 knockout mouse.This mouse is prone to develop tumors after radiation. Later it was shown that also the Period 1 protein can regulate cell cycle checkpoints [24]–[26]. Interestingly both *Period 1* and *2* are bona-fide transcriptional targets of ARNTL.

For the induction of target genes ARNTL must form a heterodimer with the CLOCK protein [28]. Target genes of the CLOCK/ARNTL heterodimer include the *Period 1*, *2* and *3* and *Cryptochromes* (*Cry 1 & 2*) [46]. The increased abundance of Period and Cryptochrome proteins [47] induces a negative feedback loop that ultimately shuts down transcription by the CLOCK/ARTNL heterodimer. When the concentration of Period and Cryptochrome decreases due to proteasomal degradation the CLOCK/ARNTL complex can initiate another round of transcription thereby completing a cycle of circadian rhythm.

Another transcriptional target of the CLOCK/ARNTL is the CDK inhibitor *p21^CIP1^* which is also regulated in a circadian manner [48]. We show here that *ARNTL* knockdown in human cells can abrogate induction of *p21^CIP1^* after p53 activation and overrides a p53-dependent cell cycle arrest. The effect on the induction of *p21^CIP1^* is in contrast with previous reports on the p21^CIP1^ regulation in *ARNTL* knockout mice [48]. In these animals *ARNTL* is required for the circadian expression of *p21^CIP1^*. This discrepancy might be explained by differential regulation of the p21^CIP1^ promoter in mice or man. In particular, this difference may arise from stress signals differences from *in vitro* versus *in vivo* conditions. Nevertheless, our data clearly indicate that there is a link between the regulation of circadian rhythm and the control of p53 activity in human cells.

### Conclusions

By screening a large scale RNAi library in human cells we have identified three novel genes that can regulate p53 function. Loss of expression for each of three genes results in a decreased ability of p53 to activate *p21^CIP1^* expression. Importantly, we showed that ARNTL is required for the p53-dependent induction of *p21^CIP1^* in two additional cell types using different ways to activate p53: a p19^ARF^-induced cell cycle arrest and a DNA damage mediated cell cycle arrest. We conclude that *ARNTL* suppression affects the ability of p53 to induce a cell cycle arrest upon cellular stress signals such as DNA damage.

## Materials and Methods

### Cell lines & culture conditions

BJtsLT cells were cultured in medium that consisted of DMEM 75% / M199 25% supplemented with 10% FCS, Penicillin and Glutamine. BJtsLT cells were cultured at 32°C in 5% CO_2_. U2OS and Phoenix cells were cultured in DMEM supplemented with 10% FCS, Penicillin and Glutamine. U2OS and Phoenix cells were cultured at 37°C in 5% CO_2_.

### Plasmids and library

Expression plasmid for ARNTL was generated by PCR from a cDNA library and subsequent cloning the PCR product into pCR3-Flag. The P19-RFP construct was described previously [11]. The construction of the library was described previously [11]. Briefly, the NKI shRNA library was designed to target 7914 human genes, using three shRNA vectors for every targeted gene, cumulating in a total of 23,742 shRNA vectors. The shRNAs are cloned into a retro-viral vector to enable infection of target cells.

### Retroviral infection

Phoenix cells were transfected using calcium phosphate method. Viral supernatant was cleared through a 0.45 µM filter. Cells were infected with the viral supernatant in presence of polybrene (8 µg/ml). The infection was repeated twice.

### shRNA barcode screen

To screen the NKI shRNA library we reasoned that we would need 100 fold coverage of the library to get a good representation of all 23,742 shRNA vectors present in the library. BJtsLT cells were infected with the NKI shRNA library. Two days after infection cells were plated at 150,000 cells/15 cm dish. In total 2×10^6^ cells were shifted to 39°C, equal number of cells were kept at 32°C. Cells at 32°C were harvested after 5 days. Cells at 39°C were harvested at 21 days after shift. From both populations gDNA was isolated using DNAzol (Invitrogen). The shRNA cassettes were amplified by PCR. The PCR product was used for in vitro RNA synthesis. RNA was labeled with Cy3 or Cy5 (Kreatech) and hybridized on a microarray. Quantification of the resulting fluorescent images was performed with Imagene 5.6 (BioDiscovery), local background was subtracted, and the data were normalized and ^2^log transformed. Additional information on barcode screens can be found at http://www.screeninc.nl.

### Colony formation assay

Cells were infected with retroviral supernatant. Two days after infection the cells were seeded at 50,000/10 cm dish and shifted to 39°C. Cells were cultured at 39°C for approx 21 days. When colonies appeared cells were fixed in MeOH/HAc (3∶1) and subsequently stained (50% MeOH/10% HAc/0.1% Coomassie).

### Western blotting

Cell lysates were separated using 10% SDS-PAAGE. Proteins were transferred to PVDF membrane and incubated with primary antibody as indicated. Primary antibodies were detected using a secondary HRP-conjugated antibody. Antibodies used for these studies: Flag (M2; Sigma), TNIP1 (1A11E3; Zymed), CDK4 (C-22; Santa Cruz), p53 (DO-1; Santa Cruz) and p21^CIP1^ (C-19; Santa Cruz).

### QRT-PCR

Total RNA was isolated using TRIzol (Invitrogen). From the total RNA cDNA was generated using Superscript II (Invitrogen) using random primers (Invitrogen). cDNA was diluted and QRT reaction was performed using Taqman probes (Applied Biosystems). All QRT reactions were run in parallel for GAPDH to control of for input cDNA. The QRT reactions were run at a AB7500 Fast Real Time PCR system (Applied Biosystems). Results shown are a representative of three independent experiments.

### p21^CIP1^ induction by γ-radiation and PXD101

For the p21^CIP1^ induction by radiation 50,000 cells were seeded per 6-well. HCT116 p53−/− cells were irradiated with 20 Gy γ-radiation from a Cs-137 source. Cells were incubated for 16 hrs and lysed. For the p21^CIP1^ induction by PXD101 50,000 cells were seeded/6-well and treated with 0.5 µM PXD101 for 16 hrs after which the cells were lysed and protein lysates were subjected to western analysis.
